# Report Available on Ensuring the Safety and Effectiveness of Laboratory Data in Electronic Health Record Systems

**Published:** 2014-05-16

Electronic health record (EHR) systems can improve patient care by making it easier to collect, share, and interpret patient data. However, variations in EHR system design, functionality, and ability to exchange data accurately (interoperability) can cause preventable patient safety risks. The Clinical Laboratory Improvement Advisory Committee (CLIAC) of the U.S. Department of Health and Human Services has raised concerns regarding the usability and interoperability of laboratory data in EHR systems. In response, in July 2012, CDC convened the Communication in Informatics Workgroup. Recommendations and suggestions from the workgroup are included in a new report from CDC, “Ensuring the Safety and Effectiveness of Laboratory Data in Electronic Health Record Systems,” available at http://www.cdc.gov/labhit.

The CDC report illustrates the seriousness of laboratory data-related interoperability issues and discrepancies in the way EHR systems display data. The report also proposes three focus areas (engagement, data integrity and usability, and innovation) for action by laboratory professionals and organizations to support development of the health information technology infrastructure and ensure the safe and effective use of laboratory information.

Actions cited within those focus areas include 1) providing laboratory expertise for health information technology decision-making in the design, development, and implementation of EHR systems; 2) guiding and maintaining data integrity and usability to ensure that laboratory data are accurately presented in the EHR and available at the point of care; and 3) partnering with stakeholders to stimulate innovation in EHR technology and usability to reduce laboratory data–related errors attributed to the use of EHR systems.

